# Crystal structure of (7-fluoro-2-oxo-2*H*-chromen-4-yl)methyl morpholine-4-carbodi­thio­ate

**DOI:** 10.1107/S2056989015021179

**Published:** 2015-11-14

**Authors:** B. R. Anitha, A. Thomas Gunaseelan, M. Vinduvahini, H. D. Kavitha, H. C. Devarajegowda

**Affiliations:** a Department of Physics, Yuvaraja’s College (Constituent College), University of Mysore, Mysore 570 005, Karnataka, India; b Department of Physics, St Philomena’s College (Autonomous), Mysore 570 015, Karnataka, India; c Department of Physics, Sri D Devaraja Urs Govt. First Grade College, Hunsur 571 105, Mysore District, Karnataka, India; d Department of Physics, Govt. Science College, Hassan 573 201, Karnataka, India

**Keywords:** crystal structure, 2*H*-chromene, morpholine-4-carbodi­thio­ate, ester, coumarin, hydrogen bonding

## Abstract

In the title compound, C_15_H_14_FNO_3_S_2_, the 2*H*-chromene ring system is close to being planar (r.m.s. deviation = 0.024 Å) and the morpholine ring adopts a chair conformation. The dihedral angle between the 2*H*-chromene ring system and the morpholine ring (all atoms) is 88.21 (11)°. In the crystal, inversion dimers linked by pairs of very weak C—H⋯F hydrogen bonds generate *R*_2_^2^(8) loops; C—H⋯O hydrogen bonds connect the dimers into [010] chains. Weak aromatic π–π stacking inter­actions between the pyran rings of the chromene systems [centroid–centroid distance = 3.6940 (16) Å] are also observed.

## Related literature   

For applications of coumarins, see: Starčević *et al.* (2011 ▸); Lodeiro *et al.* (2010 ▸); Danko *et al.* (2011 ▸). For a related structure and further synthetic details, see: Kant *et al.* (2012 ▸).
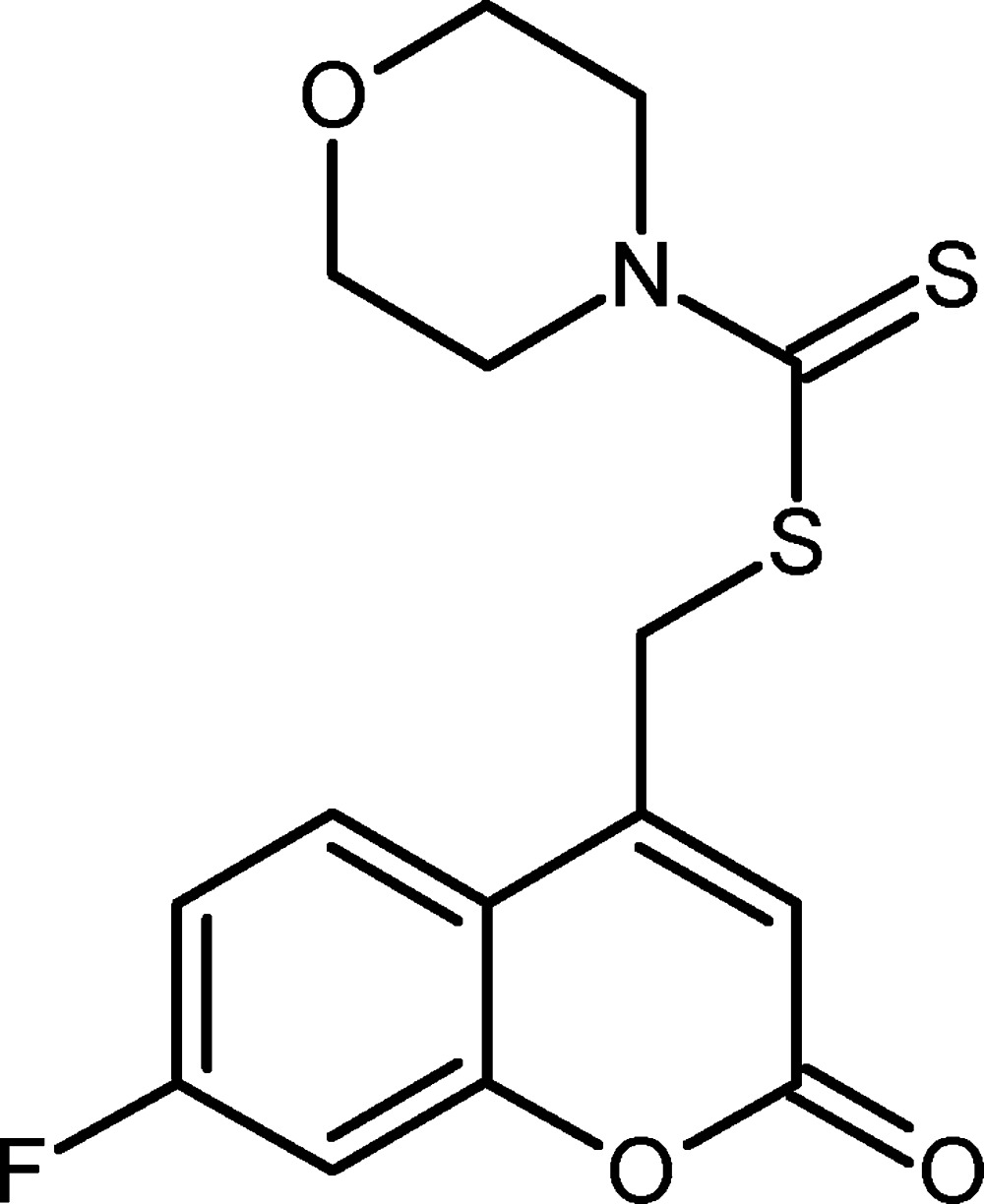


## Experimental   

### Crystal data  

C_15_H_14_FNO_3_S_2_
*M* *_r_* = 339.39
Triclinic, 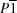

*a* = 7.0285 (3) Å
*b* = 7.8845 (3) Å
*c* = 14.8151 (6) Å
α = 74.779 (2)°
β = 87.653 (2)°
γ = 74.241 (2)°
*V* = 762.01 (5) Å^3^
*Z* = 2
Mo *K*α radiation
μ = 0.37 mm^−1^
*T* = 296 K
0.24 × 0.20 × 0.12 mm

### Data collection  

Bruker SMART CCD diffractometer
Absorption correction: multi-scan (*SADABS*; Sheldrick, 2007 ▸) *T*_min_ = 0.770, *T*_max_ = 1.000
10293 measured reflections
2683 independent reflections
1352 reflections with *I* > 2σ(*I*)
*R*_int_ = 0.171

### Refinement  

*R*[*F* ^2^ > 2σ(*F* ^2^)] = 0.048
*wR*(*F* ^2^) = 0.126
*S* = 1.09
2683 reflections
199 parameters
H-atom parameters constrained
Δρ_max_ = 0.87 e Å^−3^
Δρ_min_ = −1.15 e Å^−3^

### 

Data collection: *SMART* (Bruker, 2001 ▸); cell refinement: *SAINT* (Bruker, 2001 ▸); data reduction: *SAINT*; program(s) used to solve structure: *SHELXS97* (Sheldrick, 2008 ▸); program(s) used to refine structure: *SHELXL2014* (Sheldrick, 2015 ▸); molecular graphics: *ORTEP-3 for Windows* (Farrugia, 2012 ▸); software used to prepare material for publication: *SHELXL2014*.

## Supplementary Material

Crystal structure: contains datablock(s) I, global. DOI: 10.1107/S2056989015021179/hb7537sup1.cif

Structure factors: contains datablock(s) I. DOI: 10.1107/S2056989015021179/hb7537Isup2.hkl

Supporting information file. DOI: 10.1107/S2056989015021179/hb7537Isup3.cml

**Fig. 1.**
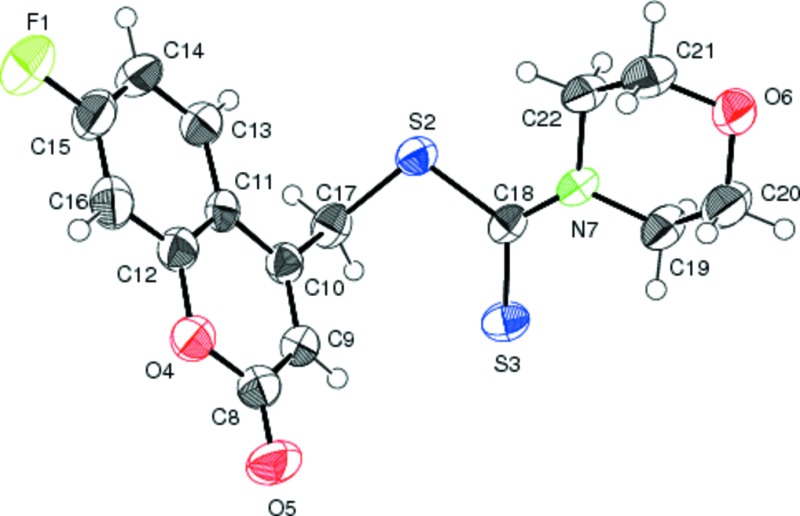
The molecular structure of the title compound. Displacement ellipsoids are drawn at the 50% probability level.

. DOI: 10.1107/S2056989015021179/hb7537fig1.tif
The mol­ecular structure of the title compound. Displacement ellipsoids are drawn at the 50% probability level.

**Fig. 2.**
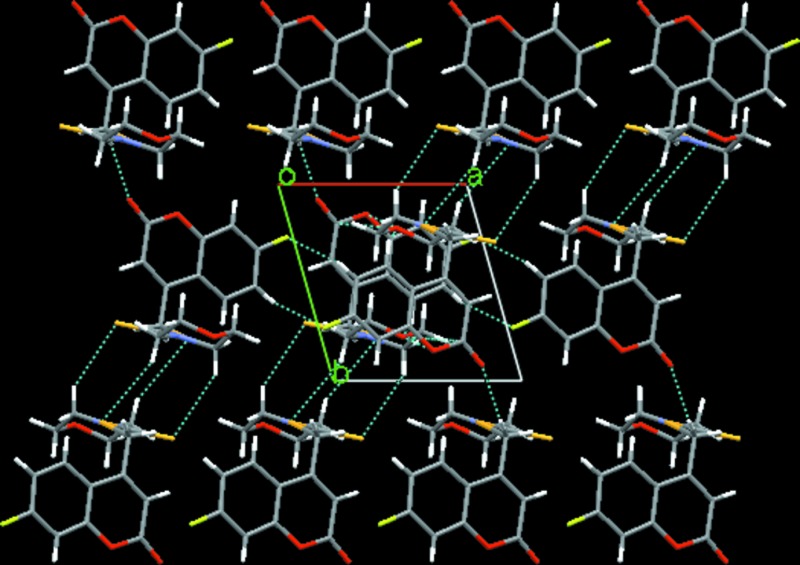
Crystal packing for the title compound with hydrogen bonds drawn as dashed lines.

. DOI: 10.1107/S2056989015021179/hb7537fig2.tif
Crystal packing for the title compound with hydrogen bonds drawn as dashed lines.

CCDC reference: 1435782

Additional supporting information:  crystallographic information; 3D view; checkCIF report

## Figures and Tables

**Table 1:** Hydrogen-bond geometry (Å, °)

*D*—H⋯*A*	*D*—H	H⋯*A*	*D*⋯*A*	*D*—H⋯*A*
C14—H14⋯F1^i^	0.93	2.56	3.451 (3)	161
C17—H17*A*⋯O5^ii^	0.97	2.45	3.346 (3)	153

Symmetry codes: (i) 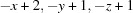; (ii) 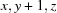.

## supplementary crystallographic information

### S1. Comment

Coumarin derivatives have many uses as antibiotics, antiviral, antimicrobial and anticoagulants agents and as pH indicators in biological systems and medical sciences (Starčević *et al.*, 2011; Lodeiro *et al.*, 2010; Danko *et al.*,2011).

The asymmetric unit of (7-fluoro-2-oxo-2*H*-chromen-4-yl)methyl morpholine-4-carbodithioate is shown in Fig. 1. The 2*H*-chromene ring systems (O4/C8–C16) is nearly planar, with a maximum deviation of 0.0591 (30) Å for atoms C8 and the morpholine ring adopts a chair conformation. The dihedral angle between best plane through the 2*H*-chromene(O4/C8–C16) ring system and the morpholine (N7\O6\C19–C22) ring is 88.21 (11)°. In the crystal, inversion-related C14—H14···F1 hydrogen bonds, forming inversion dimers with an *R*_2_^2^(8) ring motif. In the crystal, weak C—H···O hydrogen bonds (Table 1) with π–π interactions between pyran rings of chromene (C11—C16) [shortest centroid–centroid distance = 3.6940 (16) Å] are observed (Figure 2).

### S2. Experimental

The title compound was synthesized according to the reported method (Rajni Kant *et al.*, 2012). The compound is recrystallized from an ethanol-chloroform mixture as colourless needles. Yield = 77%. m.p.:413–415 K; IR (KBr, cm^-1^): 997, 1271, 1423, and 1716. GCMS: m/e: 339. 1H NMR (400 MHz, CDCl_3_, δ,.p.p.m): d 7.40 (dd, 1H, Ar—H), 7.31 (q, 1H, Ar—H), 7.24 (dd, 1H, Ar—H), 6.58 (s, 1H, Ar—H), 4.68 (s, 2H, CH_2_), 4.32 (s, 2H, CH_2_), 3.90 (s, 2H, CH_2_), 3.73 (s, 4H, CH_2_). Mol. Formula: C15H14FNO3S2. Elemental analysis: C, 53.08; H, 4.16; N, 4.13 (calculated); C, 53.12; H, 4.12; N, 4.18(found).

### S3. Refinement

All H atoms were positioned geometrically, with C—H = 0.93 Å for aromatic H, C—H = 0.97 Å for methylene H and C—H = 0.96 Å for methyl H,and refined using a riding model with *U*_iso_(H) = 1.5*U*_eq_(C) for methyl H and *U*_iso_(H) = 1.2*U*_eq_(C) for all other H.

### Crystal data

**Table:**

C_15_H_14_FNO_3_S_2_	*F*(000) = 352
*M**_r_* = 339.39	*D*_x_ = 1.479 Mg m^−^^3^
Triclinic, *P*1	Melting point: 413 K
*a* = 7.0285 (3) Å	Mo *K*α radiation, λ = 0.71073 Å
*b* = 7.8845 (3) Å	Cell parameters from 2683 reflections
*c* = 14.8151 (6) Å	θ = 1.4–25.0°
α = 74.779 (2)°	µ = 0.37 mm^−^^1^
β = 87.653 (2)°	*T* = 296 K
γ = 74.241 (2)°	Plate, colourless
*V* = 762.01 (5) Å^3^	0.24 × 0.20 × 0.12 mm
*Z* = 2	

### Data collection

**Table:**

Bruker SMART CCD diffractometer	2683 independent reflections
Radiation source: fine-focus sealed tube	1352 reflections with *I* > 2σ(*I*)
Graphite monochromator	*R*_int_ = 0.171
ω and φ scans	θ_max_ = 25.0°, θ_min_ = 1.4°
Absorption correction: multi-scan (*SADABS*; Sheldrick, 2007)	*h* = −8→8
*T*_min_ = 0.770, *T*_max_ = 1.000	*k* = −9→8
10293 measured reflections	*l* = −17→17

### Refinement

**Table:**

Refinement on *F*^2^	0 restraints
Least-squares matrix: full	Hydrogen site location: inferred from neighbouring sites
*R*[*F*^2^ > 2σ(*F*^2^)] = 0.048	H-atom parameters constrained
*wR*(*F*^2^) = 0.126	*w* = 1/[σ^2^(*F*_o_^2^) + (0.0425*P*)^2^] where *P* = (*F*_o_^2^ + 2*F*_c_^2^)/3
*S* = 1.09	(Δ/σ)_max_ = 0.001
2683 reflections	Δρ_max_ = 0.87 e Å^−^^3^
199 parameters	Δρ_min_ = −1.15 e Å^−^^3^

### Special details

**Table:**

Geometry. All e.s.d.'s (except the e.s.d. in the dihedral angle between two l.s. planes) are estimated using the full covariance matrix. The cell e.s.d.'s are taken into account individually in the estimation of e.s.d.'s in distances, angles and torsion angles; correlations between e.s.d.'s in cell parameters are only used when they are defined by crystal symmetry. An approximate (isotropic) treatment of cell e.s.d.'s is used for estimating e.s.d.'s involving l.s. planes.

### Fractional atomic coordinates and isotropic or equivalent isotropic displacement parameters (Å^2^)

**Table:**

	*x*	*y*	*z*	*U*_iso_*/*U*_eq_	
F1	0.9653 (3)	0.2708 (3)	0.50738 (15)	0.0859 (8)	
S2	0.29218 (11)	0.78840 (9)	0.14758 (6)	0.0461 (3)	
S3	−0.06235 (10)	0.70598 (9)	0.07735 (7)	0.0518 (3)	
O4	0.4232 (3)	0.1520 (2)	0.38952 (16)	0.0527 (7)	
O5	0.1885 (3)	0.0795 (2)	0.32697 (18)	0.0719 (9)	
O6	0.4365 (3)	0.7772 (3)	−0.19559 (19)	0.0646 (8)	
N7	0.2427 (3)	0.7703 (3)	−0.0227 (2)	0.0398 (8)	
C8	0.2533 (5)	0.2013 (4)	0.3352 (3)	0.0501 (11)	
C9	0.1673 (4)	0.3931 (3)	0.2956 (2)	0.0439 (10)	
H9	0.0452	0.4306	0.2640	0.053*	
C10	0.2554 (4)	0.5204 (3)	0.3023 (2)	0.0388 (9)	
C11	0.4419 (4)	0.4640 (3)	0.3540 (2)	0.0391 (9)	
C12	0.5198 (4)	0.2788 (3)	0.3958 (2)	0.0428 (9)	
C13	0.5487 (5)	0.5827 (4)	0.3661 (2)	0.0506 (10)	
H13	0.4997	0.7074	0.3394	0.061*	
C14	0.7248 (5)	0.5187 (4)	0.4167 (3)	0.0581 (11)	
H14	0.7959	0.5983	0.4237	0.070*	
C15	0.7935 (5)	0.3342 (5)	0.4566 (3)	0.0576 (11)	
C16	0.6958 (4)	0.2118 (4)	0.4476 (2)	0.0540 (11)	
H16	0.7458	0.0876	0.4754	0.065*	
C17	0.1662 (4)	0.7184 (3)	0.2536 (2)	0.0455 (10)	
H17A	0.1744	0.7928	0.2953	0.055*	
H17B	0.0276	0.7377	0.2387	0.055*	
C18	0.1546 (4)	0.7529 (3)	0.0590 (2)	0.0358 (9)	
C19	0.1388 (5)	0.7751 (4)	−0.1081 (3)	0.0513 (11)	
H19A	0.0697	0.9005	−0.1387	0.062*	
H19B	0.0410	0.7068	−0.0910	0.062*	
C20	0.2771 (5)	0.6969 (4)	−0.1743 (3)	0.0647 (12)	
H20A	0.3295	0.5666	−0.1473	0.078*	
H20B	0.2047	0.7139	−0.2318	0.078*	
C21	0.5445 (4)	0.7488 (4)	−0.1116 (3)	0.0568 (11)	
H21A	0.6569	0.7993	−0.1262	0.068*	
H21B	0.5948	0.6187	−0.0837	0.068*	
C22	0.4230 (4)	0.8337 (4)	−0.0435 (3)	0.0468 (10)	
H22A	0.3857	0.9654	−0.0683	0.056*	
H22B	0.5005	0.8042	0.0140	0.056*	

### Atomic displacement parameters (Å^2^)

**Table:**

	*U*^11^	*U*^22^	*U*^33^	*U*^12^	*U*^13^	*U*^23^
F1	0.0668 (14)	0.1122 (16)	0.077 (2)	−0.0272 (13)	−0.0263 (14)	−0.0140 (14)
S2	0.0509 (5)	0.0439 (4)	0.0471 (9)	−0.0193 (4)	−0.0053 (5)	−0.0104 (4)
S3	0.0364 (5)	0.0581 (5)	0.0627 (9)	−0.0154 (4)	−0.0008 (5)	−0.0158 (5)
O4	0.0643 (14)	0.0439 (11)	0.049 (2)	−0.0201 (11)	−0.0109 (13)	−0.0038 (11)
O5	0.0950 (19)	0.0548 (12)	0.077 (2)	−0.0418 (14)	−0.0197 (16)	−0.0102 (12)
O6	0.0576 (16)	0.1042 (16)	0.047 (2)	−0.0387 (13)	0.0110 (14)	−0.0294 (15)
N7	0.0360 (15)	0.0413 (13)	0.047 (3)	−0.0110 (11)	−0.0068 (14)	−0.0177 (14)
C8	0.063 (2)	0.0512 (18)	0.043 (3)	−0.0255 (18)	0.0008 (19)	−0.0129 (17)
C9	0.0493 (18)	0.0480 (16)	0.037 (3)	−0.0183 (14)	−0.0009 (17)	−0.0094 (15)
C10	0.0443 (17)	0.0392 (14)	0.034 (3)	−0.0112 (13)	0.0029 (16)	−0.0113 (14)
C11	0.0480 (18)	0.0463 (16)	0.027 (3)	−0.0158 (14)	0.0042 (17)	−0.0140 (15)
C12	0.0501 (19)	0.0433 (16)	0.038 (3)	−0.0154 (15)	0.0008 (17)	−0.0130 (15)
C13	0.059 (2)	0.0541 (17)	0.044 (3)	−0.0217 (16)	0.0030 (19)	−0.0158 (17)
C14	0.060 (2)	0.076 (2)	0.054 (3)	−0.0350 (19)	−0.001 (2)	−0.025 (2)
C15	0.048 (2)	0.081 (2)	0.046 (3)	−0.0198 (19)	−0.006 (2)	−0.019 (2)
C16	0.054 (2)	0.0603 (19)	0.041 (3)	−0.0108 (17)	−0.001 (2)	−0.0068 (18)
C17	0.0507 (19)	0.0425 (14)	0.047 (3)	−0.0081 (14)	−0.0015 (18)	−0.0221 (15)
C18	0.0373 (16)	0.0292 (12)	0.040 (3)	−0.0037 (12)	−0.0061 (16)	−0.0115 (14)
C19	0.0430 (19)	0.0588 (18)	0.057 (3)	−0.0119 (16)	−0.0049 (19)	−0.0240 (18)
C20	0.055 (2)	0.087 (2)	0.067 (4)	−0.030 (2)	0.001 (2)	−0.036 (2)
C21	0.0397 (19)	0.0622 (19)	0.074 (4)	−0.0163 (16)	0.000 (2)	−0.024 (2)
C22	0.0393 (17)	0.0498 (16)	0.058 (3)	−0.0168 (14)	−0.0002 (18)	−0.0195 (17)

### Geometric parameters (Å, º)

**Table:**

F1—C15	1.350 (3)	C12—C16	1.382 (4)
S2—C18	1.783 (3)	C13—C14	1.373 (4)
S2—C17	1.804 (3)	C13—H13	0.9300
S3—C18	1.660 (3)	C14—C15	1.374 (4)
O4—C8	1.374 (3)	C14—H14	0.9300
O4—C12	1.375 (3)	C15—C16	1.362 (4)
O5—C8	1.203 (3)	C16—H16	0.9300
O6—C20	1.417 (3)	C17—H17A	0.9700
O6—C21	1.419 (4)	C17—H17B	0.9700
N7—C18	1.329 (4)	C19—C20	1.484 (4)
N7—C19	1.473 (4)	C19—H19A	0.9700
N7—C22	1.478 (3)	C19—H19B	0.9700
C8—C9	1.437 (4)	C20—H20A	0.9700
C9—C10	1.340 (3)	C20—H20B	0.9700
C9—H9	0.9300	C21—C22	1.472 (4)
C10—C11	1.446 (4)	C21—H21A	0.9700
C10—C17	1.504 (3)	C21—H21B	0.9700
C11—C12	1.390 (3)	C22—H22A	0.9700
C11—C13	1.398 (4)	C22—H22B	0.9700
			
C18—S2—C17	104.01 (14)	C10—C17—S2	111.30 (19)
C8—O4—C12	121.2 (2)	C10—C17—H17A	109.4
C20—O6—C21	108.9 (3)	S2—C17—H17A	109.4
C18—N7—C19	121.1 (2)	C10—C17—H17B	109.4
C18—N7—C22	124.6 (3)	S2—C17—H17B	109.4
C19—N7—C22	112.4 (3)	H17A—C17—H17B	108.0
O5—C8—O4	116.7 (3)	N7—C18—S3	124.0 (3)
O5—C8—C9	126.3 (3)	N7—C18—S2	113.0 (2)
O4—C8—C9	117.0 (3)	S3—C18—S2	123.0 (2)
C10—C9—C8	122.8 (3)	N7—C19—C20	111.8 (3)
C10—C9—H9	118.6	N7—C19—H19A	109.2
C8—C9—H9	118.6	C20—C19—H19A	109.2
C9—C10—C11	119.0 (3)	N7—C19—H19B	109.2
C9—C10—C17	120.7 (3)	C20—C19—H19B	109.2
C11—C10—C17	120.3 (2)	H19A—C19—H19B	107.9
C12—C11—C13	117.5 (3)	O6—C20—C19	112.9 (3)
C12—C11—C10	117.9 (2)	O6—C20—H20A	109.0
C13—C11—C10	124.6 (3)	C19—C20—H20A	109.0
O4—C12—C16	116.1 (2)	O6—C20—H20B	109.0
O4—C12—C11	121.7 (3)	C19—C20—H20B	109.0
C16—C12—C11	122.1 (3)	H20A—C20—H20B	107.8
C14—C13—C11	121.2 (3)	O6—C21—C22	112.4 (3)
C14—C13—H13	119.4	O6—C21—H21A	109.1
C11—C13—H13	119.4	C22—C21—H21A	109.1
C15—C14—C13	118.5 (3)	O6—C21—H21B	109.1
C15—C14—H14	120.7	C22—C21—H21B	109.1
C13—C14—H14	120.7	H21A—C21—H21B	107.9
F1—C15—C16	118.2 (3)	C21—C22—N7	111.6 (2)
F1—C15—C14	118.8 (3)	C21—C22—H22A	109.3
C16—C15—C14	123.0 (3)	N7—C22—H22A	109.3
C15—C16—C12	117.6 (3)	C21—C22—H22B	109.3
C15—C16—H16	121.2	N7—C22—H22B	109.3
C12—C16—H16	121.2	H22A—C22—H22B	108.0
			
C12—O4—C8—O5	173.5 (3)	F1—C15—C16—C12	179.7 (3)
C12—O4—C8—C9	−8.1 (5)	C14—C15—C16—C12	0.0 (6)
O5—C8—C9—C10	−175.2 (3)	O4—C12—C16—C15	−178.9 (3)
O4—C8—C9—C10	6.6 (5)	C11—C12—C16—C15	−0.3 (5)
C8—C9—C10—C11	−2.1 (5)	C9—C10—C17—S2	−101.8 (3)
C8—C9—C10—C17	175.5 (3)	C11—C10—C17—S2	75.8 (4)
C9—C10—C11—C12	−0.9 (5)	C18—S2—C17—C10	92.2 (2)
C17—C10—C11—C12	−178.5 (3)	C19—N7—C18—S3	9.0 (3)
C9—C10—C11—C13	179.9 (3)	C22—N7—C18—S3	172.21 (18)
C17—C10—C11—C13	2.3 (6)	C19—N7—C18—S2	−170.17 (18)
C8—O4—C12—C16	−176.0 (3)	C22—N7—C18—S2	−7.0 (3)
C8—O4—C12—C11	5.4 (5)	C17—S2—C18—N7	−170.21 (18)
C13—C11—C12—O4	178.5 (3)	C17—S2—C18—S3	10.60 (18)
C10—C11—C12—O4	−0.7 (5)	C18—N7—C19—C20	−149.2 (3)
C13—C11—C12—C16	0.1 (5)	C22—N7—C19—C20	45.7 (3)
C10—C11—C12—C16	−179.2 (3)	C21—O6—C20—C19	59.9 (4)
C12—C11—C13—C14	0.5 (5)	N7—C19—C20—O6	−53.0 (4)
C10—C11—C13—C14	179.7 (3)	C20—O6—C21—C22	−61.1 (3)
C11—C13—C14—C15	−0.9 (6)	O6—C21—C22—N7	55.4 (4)
C13—C14—C15—F1	−179.1 (3)	C18—N7—C22—C21	148.5 (3)
C13—C14—C15—C16	0.6 (6)	C19—N7—C22—C21	−47.0 (3)

### Hydrogen-bond geometry (Å, º)

**Table:**

*D*—H···*A*	*D*—H	H···*A*	*D*···*A*	*D*—H···*A*
C14—H14···F1^i^	0.93	2.56	3.451 (3)	161
C17—H17*A*···O5^ii^	0.97	2.45	3.346 (3)	153

Symmetry codes: (i) −*x*+2, −*y*+1, −*z*+1; (ii) *x*, *y*+1, *z*.
