# Supplementary figures and images for: Crystal structure of (7-fluoro-2-oxo-2H-chromen-4-yl)methyl morpholine-4-carbodi­thio­ate
Source: Acta Crystallogr E Crystallogr Commun. 2015 Nov 14;71(Pt 12):o928–9. doi: 10.1107/S2056989015021179 (PMC4719885; doi:10.1107/S2056989015021179)

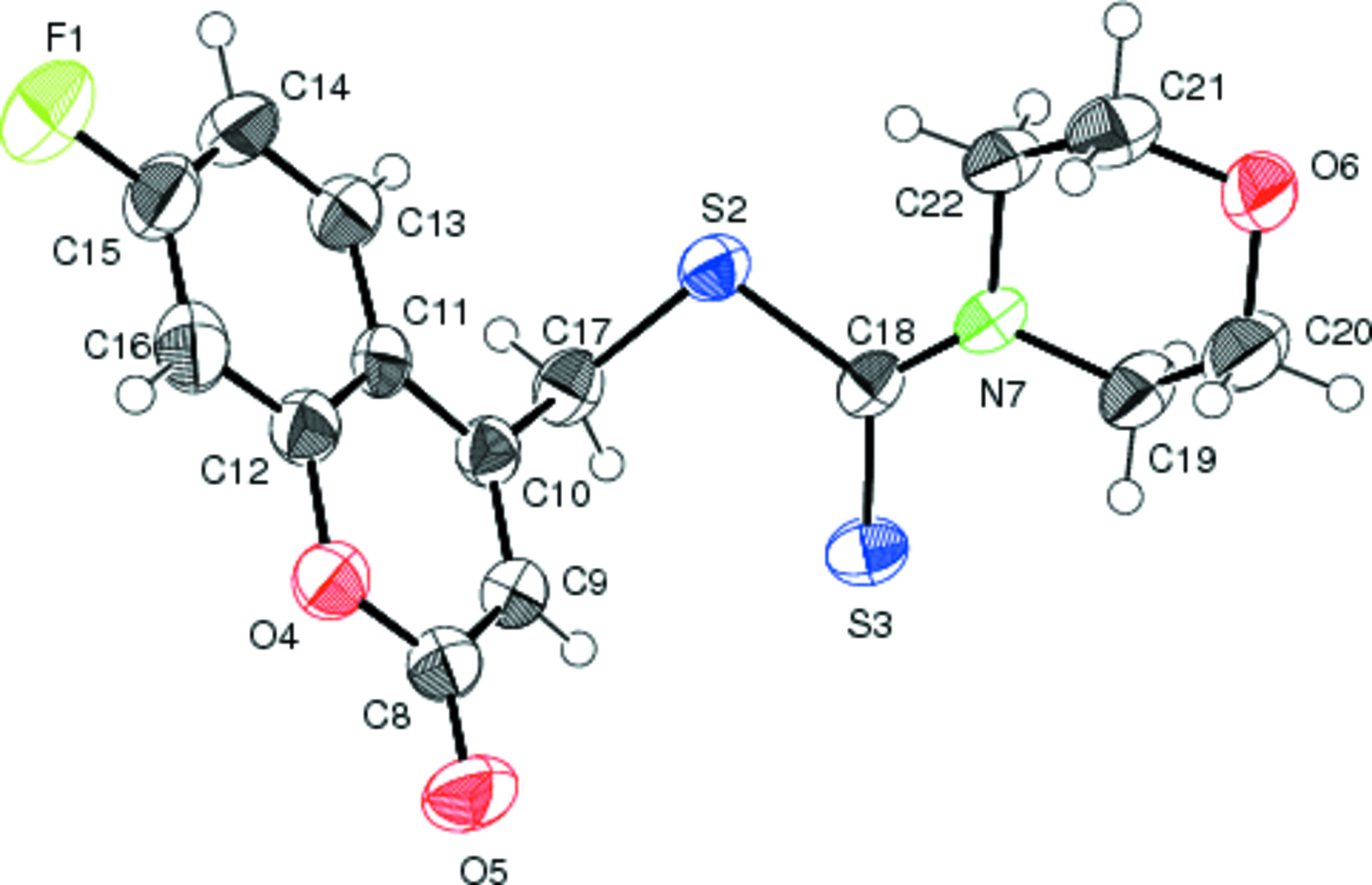

Supplement: Supplementary file 4 [file e-71-0o928-fig1.tif]

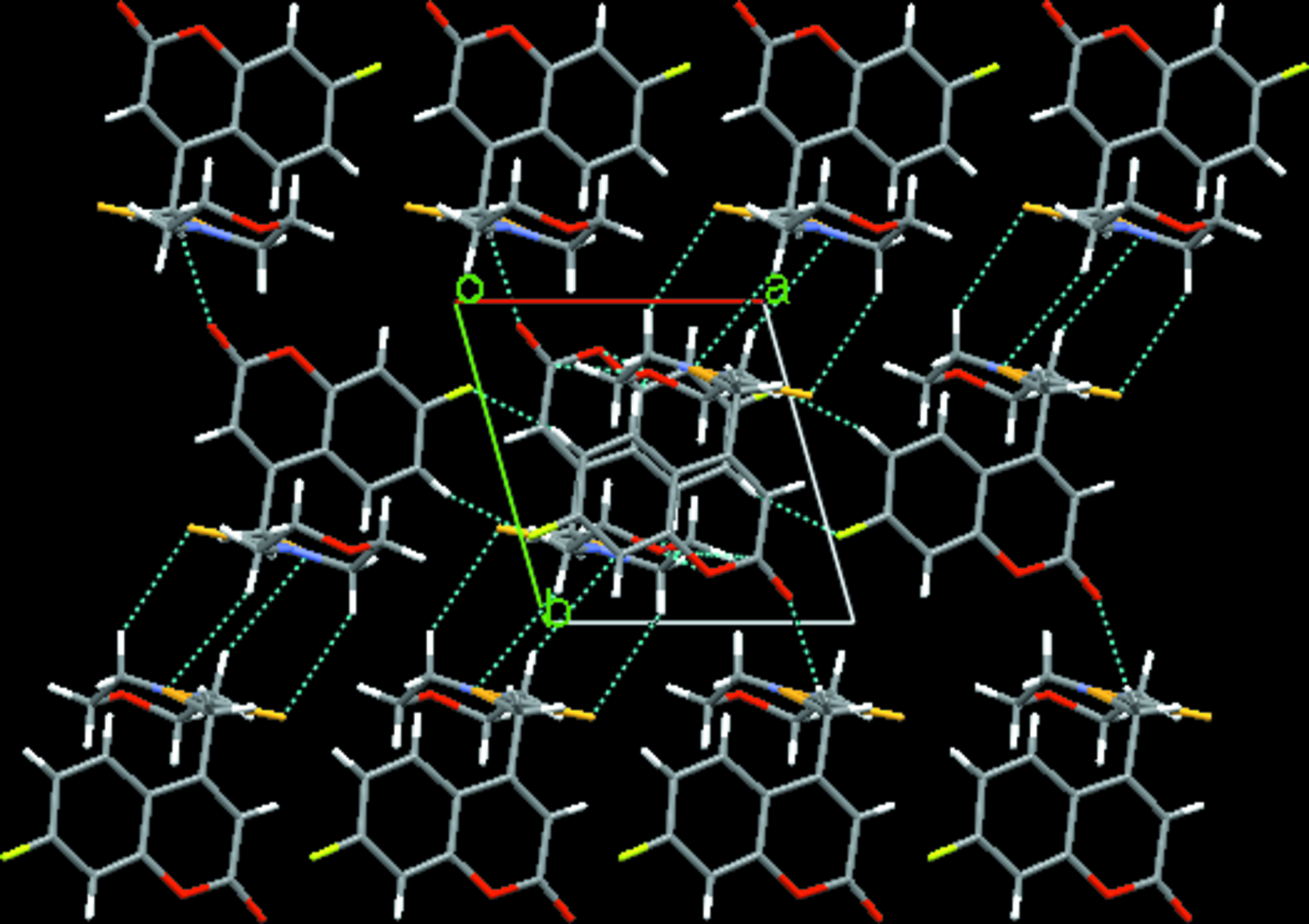

Supplement: Supplementary file 5 [file e-71-0o928-fig2.tif]
